# Grafting of short elastin-like peptides using an electric field

**DOI:** 10.1038/s41598-022-21672-9

**Published:** 2022-11-04

**Authors:** Nuttanit Pramounmat, Sogol Asaei, Jacob D. Hostert, Kathleen Young, Horst A. von Recum, Julie N. Renner

**Affiliations:** 1 Department of Chemical and Biomolecular Engineering, Case Western Reserve University, Cleveland, USA; 2 Department of Biomedical Engineering, Case Western Reserve University, Cleveland, USA

**Keywords:** Biomaterials, Molecular engineering

## Abstract

Surface-grafted elastin has found a wide range of uses such as sensing, tissue engineering and capture/release applications because of its ability to undergo stimuli-responsive phase transition. While various methods exist to control surface grafting in general, it is still difficult to control orientation as attachment occurs. This study investigates using an electric field as a new approach to control the surface-grafting of short elastin-like polypeptide (ELP). Characterization of ELP grafting to gold via quartz crystal microbalance with dissipation, atomic force microscopy and temperature ramping experiments revealed that the charge/hydrophobicity of the peptides, rearrangement kinetics and an applied electric field impacted the grafted morphology of ELP. Specifically, an ELP with a negative charge on the opposite end of the surface-binding moiety assembled in a more upright orientation, and a sufficient electric field pushed the charge away from the surface compared to when the same peptide was assembled in no electric field. In addition, this study demonstrated that assembling charged ELP in an applied electric field impacts transition behavior. Overall, this study reveals new strategies for achieving desirable and predictable surface properties of surface-bound ELP.

## Introduction

Surface functionalization with biomolecules is important for the development of next-generation dynamic or smart biointerfaces^1^. Biomolecules can be designed to be multifunctional, allowing for easy attachment to inorganic surfaces and control of surface properties. Specifically, dynamic biointerfaces are surfaces with the capacity to respond to external stimuli reversibly or irreversibly. The ability to control interfaces on-demand at the molecular level aids in the development of scaffolds for antifouling materials, tissue engineering, drug delivery platforms, and biosensors^2^. One way to achieve stimuli-responsiveness is by utilizing surface-bound thermoresponsive polymers, which have been utilized for cell culture and capture/release applications^3,4^. Biosensor development is another growing field where thermoresponsive polymers play essential roles in detecting molecules of interest^4–6^.

Elastin-like polypeptides are stimuli-responsive biopolymers that undergo a transition from soluble to insoluble with ionic strength^7,8^, temperature^9^, pH^10^, pressure^11^, and concentration^12^ acting as stimuli. The conformation is understood to be extended when it is soluble and collapsed when it undergoes transition^13,14^. Elastin is beneficial as a stimuli-responsive biomolecule because the transition (often quantified as a transition temperature, or *T*_t_) can be easily and predictably modulated by tuning the sequence hydrophobicity via a guest residue^15,16^. Surface-grafted elastin has found many applications including in switchable interfaces^17–19^, vascular engineering^20,21^, and controlling electrode architecture^22,23^. Surface-grafting of elastin-like polypeptides has been facilitated by amine-reactive crosslinker chemistry with N-hydroxysuccinimide^24^, thiol binding to gold^25–27^, affinity sequences^28,29^, and physical adsorption^30,31^. All the listed methods involve incubation of elastin-like polypeptide molecules near the surface of interest, leaving the interaction between elastin-like polypeptides and the surfaces uncontrolled and the subsequent properties often unpredictable.

One way to control grafting is by using an electric field which is advantageous because no additional chemistry is required, and it is simple to control. Electric fields have been used to control the assembly and grafting density of alkanethiol monolayers^32–36^, as well as biomolecules such as DNA^37–40^ and peptides^41–44^. Recently, Gibson and Mendes demonstrated that an electric field influences the adsorption kinetics, orientation, and stability of charged oligopeptides on gold^42^. Specifically, it was shown that the presence of an electric field (positive or negative) slowed adsorption of a charged oligopeptide. However, no kinetic modeling was performed, and thus the quantitative impact of electric fields on peptide adsorption and rearrangement remains uncharacterized. While grafting density did not appear to be impacted in the work by Gibson and Mendes, other work indicates electric fields impact the grafting density of monolayers^45^. It is known that the grafting approach impacts the thermoresponsivity as well as the grafting density of surface-bound elastin-like polypeptides^24^, and other thermoresponsive polymers such as poly(glycidyl ether)^46^.

Control of ELP grafting via electric field remains unexplored, and while many studies focus on longer ELP^24^, shorter sequences have distinct advantages including the ability to precisely control the amino acid content and generate new sequences synthetically relatively quickly. In this work, we investigate using an electric-field to control surface-grafting of short elastin-like polypeptide (ELP) for the first time. Specifically, the goal of this study is to test the hypothesis that electric field and ELP composition will impact the ELP grafting density, grafting kinetics, grafted morphology and structural transition properties. In doing so, we uncover specific drivers for ELP assembly that can be used to control ELP surface properties for future use in applications such as sensing, tissue engineering and capture/release applications.

## Results and discussion

### Peptide design

Peptides were designed to have (1) a cysteine amino acid on the N-terminus for attachment to the gold surface, (2) five-repeats of the ELP domain (VPGVG) and 3) a 2-aminobenzoyl (Abz) and 2,4-dinitrophenyl (Dnp) fluorescence resonance energy transfer (FRET) pair for the detection of ELP structural change in solution in response to temperature and salt concentration. The N-terminal charge of the peptide was shielded by the placement of an Abz fluorophore. The Dnp quencher was placed on a functional group of a lysine guest residue placed on the C-terminus of the peptide. This FRET pair was selected due to the relatively small size of the fluorophores so the impact on ELP behavior would be minimized. It was expected FRET could serve as a high throughput way to confirm phase transition behavior and has been demonstrated previously for elastin as a temperature sensor^47,48^. The guest residue (X, where X is any amino acid except proline) of the ELP domain (VPGXG) was chosen to be valine (V) because it would lead to a transition temperature of the ELP within the range of clinical applications due to its high hydrophobicity^15^. Valine was also chosen because it has been observed that short ELP sequences (less than 5 repeats) with V in the guest residue display reversible transition behavior when attached to gold nanoparticles^49,50^. Therefore, it was expected that our peptide grafted to gold would exhibit similar behavior. To make an uncharged ELP, the C-terminus was amidated. To make a negatively (−) charged ELP (i.e., (−) charged ELP), the C-terminus was left unmodified. **Table ****1** provides the full amino acid sequences of the short elastin peptides chemically synthesized for this study.

**Table 1:** The ELP sequences designed for this study consist of uncharged ELP and (−)charged ELP at neutral pH. Both peptides have five repeats of the VPGVG pentapeptide sequence, 2-aminobenzoyl (Abz) followed by cysteine amino acid at the N-terminus, and 2, 4-dinitrophenyl (Dnp) attached to the amine functional group on lysine (K) before the C-terminus.

Peptide name	Molecular weight	Peptide sequence
Uncharged ELP	2580.98	Abz-CVPGVGVPGVGVPGVGVPGVGVPGVGK(Dnp)-Amidated
(−)Charged ELP	2581.96	Abz-CVPGVGVPGVGVPGVGVPGVGVPGVGK(Dnp)

### Transition behavior and aggregation measured via DLS

**Figure 1:**
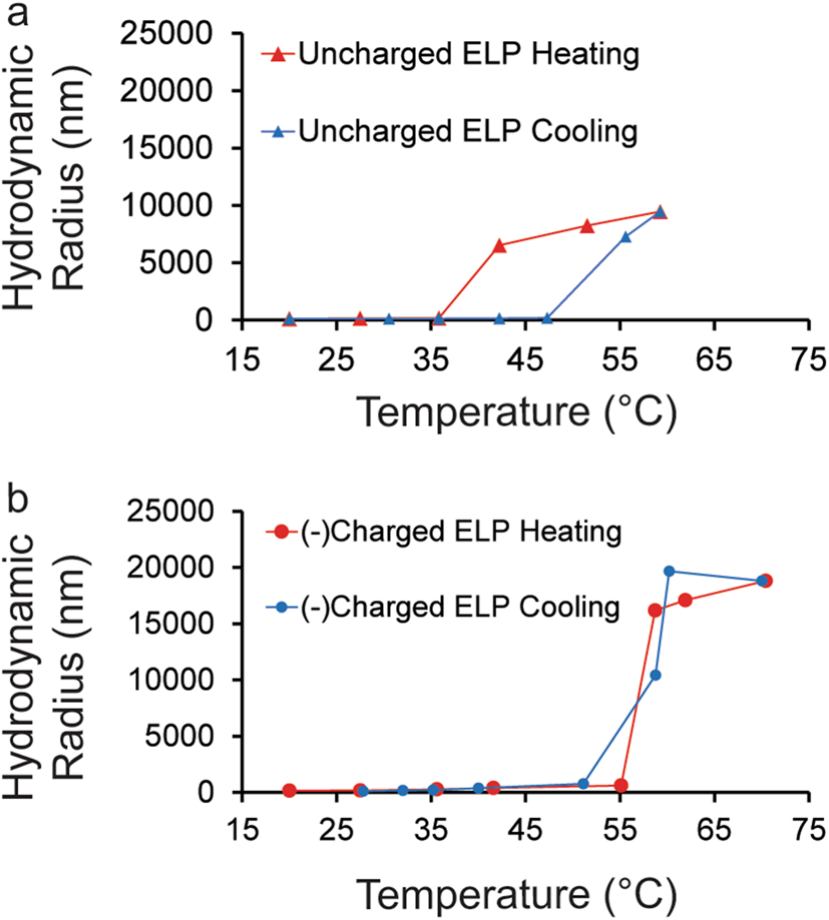
Uncharged ELP (a, data represented as triangles) and (−)charged ELP (b, data represented as circles) in DI water display reversible transition behavior. The average hydrodynamic radius of three accumulations is measured as the temperature of 10 µg/mL solutions of ELP is raised from 20 °C to 60 °C and 70 °C respectively (heating data shown in red) and cooled back down to 20 °C and 27 °C respectively (cooling data shown in blue) at a rate of 2 °C per minute. Lines between data points are added to help the reader more clearly see the transition behavior.

First, reversible phase transition behavior of the peptides was confirmed using dynamic light scattering. Hydrodynamic radius (R_h_) increased as 0.01 mg/mL solutions in DI water were heated from 20 °C to 60 °C and 70 °C for uncharged (Fig. 1a, data shown in red triangles) and (−)charged ELP (Fig. 1b,data shown in red circles) respectively at 2 °C per minute. Solutions of uncharged (Fig. 1a, data shown in blue triangles) and (−)charged (Fig. 1b, data shown in blue circles) ELP were cooled at the same rate. The sharp increase in R_h_ as the solutions were heated indicated a transition of the ELP occurred. The transition temperature was described as the temperature halfway between dramatically low and high R_h_ upon heating or cooling. For heating, the transition temperature was observed in the solution of uncharged and (−)charged ELP around ~ 39 °C and ~ 57 °C, respectively. For cooling, the transition temperature was observed in the solution of uncharged and (−)charged ELP around ~ 51 °C and ~ 56 °C, respectively. The higher transition temperature of (−)charged ELP was anticipated due to the higher hydrophilicity that was introduced by its charged C-terminus^14^. More pronounced hysteresis occurred with the uncharged ELP as seen in Fig. 1a during cooling. Literature indicates hysteresis is caused by non-equilibrium states and can manifest in a wide range of behaviors which depend on factors such as chain length, heating rate, and the strength of peptide-peptide interactions^51^. These results from DLS demonstrating phase transition behavior were corroborated by FRET measurements shown in Fig. S1 and Fig. S2 where at 0.05 mg/mL transition behavior is observed in uncharged ELP between 20 and 40 °C upon heating, but the transition of charged ELP occurs outside of the temperature range tested using FRET (up to 45 °C). FRET measurements also revealed the expected trends with hydrophobicity, salt and concentration for phase transition behavior as explained in the SI text.

Next, DLS was used to confirm that 0.5 M NaCl did not cause the peptides to transition at the grafting conditions described in the next Sect. (0.01 mg/mL peptide in DI water grafted at 20 °C). DLS revealed that the R_h_ for uncharged ELP and (−)charged ELP at 20 °C and 0.01 mg/mL in DI water with 0.5 M NaCl were ~ 240 and ~ 310 nm respectively, which is comparable to the sizes measured in DI water below the transition temperature. Compared to the R_h_ when both peptides undergo phase transition due to temperature, the size is two orders of magnitude smaller.

Overall, the characterization of ELP in solution using DLS and FRET verified that the expected reversible stimuli-responsive behavior was present in the designed peptides. In the next section, the hypothesis that an electric field can control the surface grafting of ELP was explored.

### Impact of electric field on hydrated mass loading and molecular orientation

**Figure 2:**
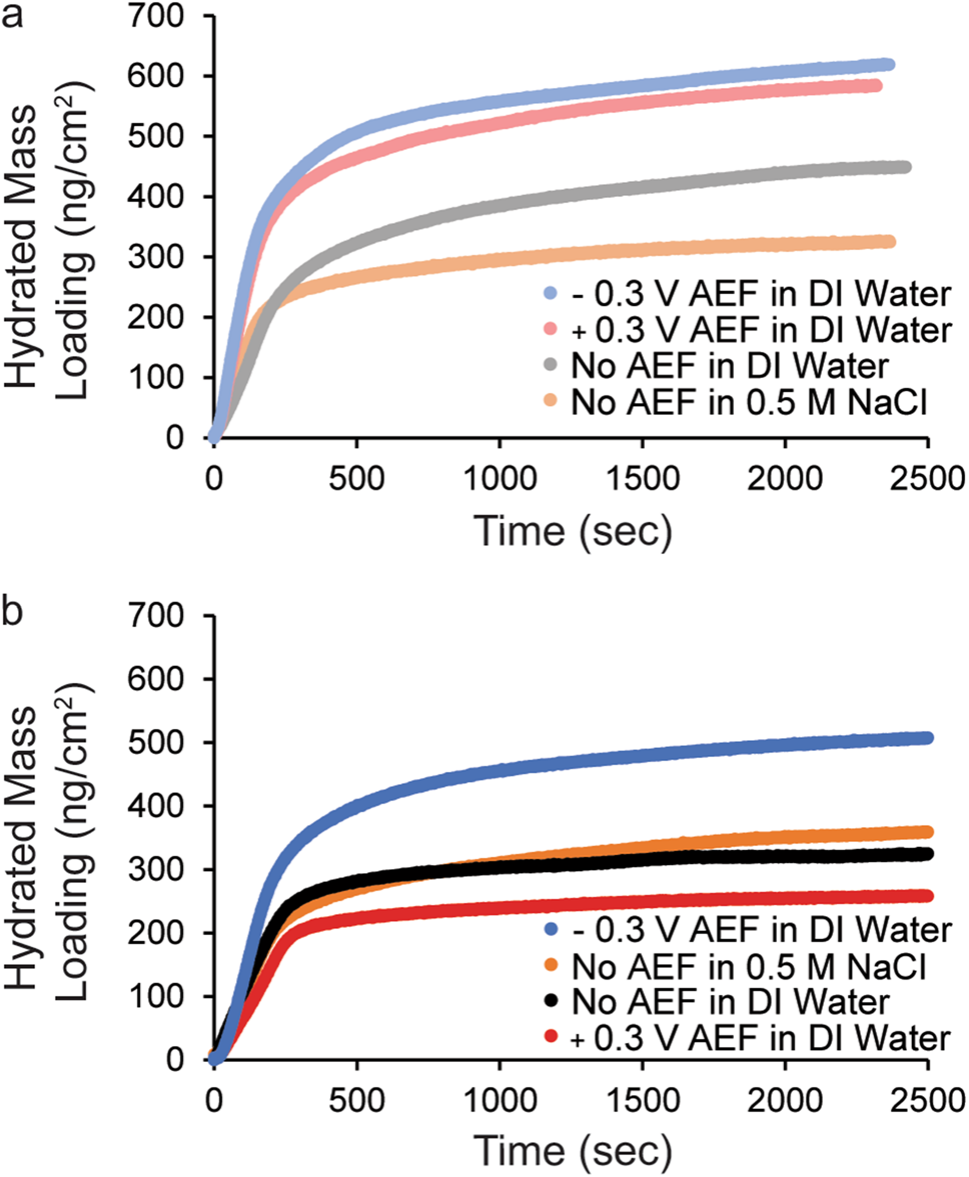
Assembly of charged ELP in an electric field impacts hydrated mass loading. Hydrated mass loading is monitored with time by a quartz crystal microbalance with dissipation in an electrochemical module at 20 °C as (**a**) uncharged ELP and (**b**) (−)charged ELP is assembled at four grafting conditions: (1) − 0.3 V AEF in DI water (light blue for uncharged, blue for (−)charged); (2) + 0.3 V AEF in water (light red for uncharged, red for (−)charged); (3) no AEF in water (grey for uncharged, black for (−)charged); and (4) no AEF in 0.5 M NaCl (light orange for uncharged, orange for (-)charged). All voltages are vs. a Ag/AgCl reference. Recall that the potential difference measured between the working and counter electrode, which generates the field, is − 1.0 V to − 1.1 V at − 0.3 V vs. Ag/AgCl and is − 0.2 V to − 0.3 V at + 0.3 V vs. Ag/AgCl.

The grafting of ELP onto a gold surface was conducted in a quartz crystal microbalance with dissipation using an electrochemical module. Hydrated mass loading (reported in Fig. 2 in ng/cm^2^) was monitored for at least 40 min at four different grafting conditions for each ELP: without applied electric field (no AEF) in DI water, at − 0.3 V AEF vs. Ag/AgCl in DI water, at + 0.3 V AEF vs. Ag/AgCl in DI water, and no AEF in 0.5 M NaCl. All experiments were carried out at a controlled temperature of 20 °C. The voltage between the gold working electrode and the platinum counter electrode and the distance between the two plates dictates electric field strength. In this experiment, the voltage across the two plates was approximately − 1.0 to − 1.1 V during DI water baseline at − 0.3 V vs. Ag/AgCl in DI water. The distance between the two plates is 0.8 mm, making the approximate applied electric field strength 1300–1400 V/m at − 0.3 V vs. Ag/AgCl. The voltage across the two plates at + 0.3 V vs. Ag/AgCl in DI water was approximately − 0.2 to − 0.3 V vs. Ag/AgCl, making the approximate applied electric field strength 300–400 V/m. Therefore, in both cases the field would cause a negative charge to migrate opposite the working electrode toward the counter electrode, with − 0.3 V vs. Ag/AgCl resulting in a stronger field than + 0.3 V vs. Ag/AgCl. The experimental groups in this manuscript are labeled with the voltage vs. Ag/AgCl reference as the setting to apply the electric field.

At 40 min, the AEFs increased the mass uptake of the uncharged ELP from 450 to ~ 590 ng/cm^2^ on a gold surface compared to no AEF (Fig. 2a). Previous studies indicate that an applied positive potential vs. a Ag/AgCl reference electrode impacts the packing density of alkane thiols during adsorption, presumably via enhancing the electron transfer process between sulfur and gold surface^33–35^, and thus may also impact the packing density of the peptides in this study. Other studies have observed increases in packing density of a thioctic acid derivative when negative potentials were applied and suggested a potential-dependent surface reaction with no electron transfer was occurring^36^. Interestingly, in a highly related study of the adsorption of cysteine-terminated positively charged oligopeptides under similar AEF conditions (between − 0.4 V and + 0.3 V vs. Ag/AgCl) in PBS, the AEF had no impact on packing density^42^. Since our study is carried out in DI water instead of PBS, higher water coverage on the surface could cause some of the difference in adsorption behavior seen between this and the aforementioned work.

For the (−)charged ELP, a different trend was observed in the hydrated mass values at 40 min (Fig. 2b). The + 0.3 V vs. Ag/AgCl AEF condition had a similar low loading of the peptide compared to no AEF, while a − 0.3 V vs. Ag/AgCl AEF condition increased loading. It is expected that the negative potential forces the (−)charged C-terminus to be oriented away from the gold surface while the cysteine-attached N-terminus is in contact with the gold surface. Electric fields have been shown to impact molecular orientation in simulations of alkanethiols on gold^45^, as well as experiments with charged oligopeptides^42,43,53,54^.

The result that a sufficiently high AEF directly impacts the hydrated mass loading as charged ELP is grafted to gold supports the premise that electric fields have the capacity to serve as an effective technique to control the loading and orientation designed peptides on solid surfaces by the attraction and repulsion of charged moieties. Experiments with peptides containing five positively charged lysine residues revealed that the electric field influenced orientation but not loading, likely due to the high charge repulsion of the many positively charged residues^42^. Our results indicate that the charge repulsion is possibly reduced when using one terminally located charge, thus minimizing the counteracting effect of charge repulsion observed in that study. As expected, charge did have some impact on loading because it was observed that in DI water charged peptide had lower overall hydrated mass density compared to uncharged peptide without an AEF. This observation may be due to charge repulsion or potentially an interaction between the (−)charged (deprotonated) carboxyl group with the gold surface. Fourier transform infrared (FTIR) spectra taken on QCM-D sensors with assembled peptide samples shown Fig. 2a and 2b confirm the presence of ELP, and the orientation of the carboxyl group in the peptide layer (Fig. S3-I and II) as discussed in the SI. To further confirm the molecular orientation was impacted, thickness estimates of all samples assembled in Fig. 2a and 2b were completed in a dry environment using spectroscopic ellipsometry (Fig. S4), also discussed in the SI.

To understand the impact of salt on grafting and charge shielding, charged and uncharged ELP were grafted onto a gold surface without an AEF in 0.5 M NaCl. DLS results discussed in the previous section confirmed that 0.5 M NaCl did not induce transition of the peptides in solution at 10 µg/mL peptide concentration. The mass loading of the uncharged ELP decreased in the presence of 0.5 M NaCl, while the mass loading of the (−)charged peptide was similar with and without the addition of 0.5 M NaCl. These behaviors may be attributed to higher water coverage in DI water versus salt-containing solutions as mentioned previously. In the case of (−)charged ELP in the presence of salt, the water coverage effect may be counterbalanced by charge shielding. The presence of salt also provided an opportunity to observe the impact of charge shielding on grafting mechanism, discussed in the next section.

### Kinetic study of ELP grafting

A kinetic analysis of the grafting results shown in Fig. 2 was carried out by fitting a biexponential model to the frequency shift with time measured by quartz crystal microbalance with dissipation. The first step of this model accounts for reversible binding of the ELP to the gold surface via a thiol-gold interaction through a cysteine residue (Eq. 1). We postulate the ELP will bind to accessible gold sites in any conformation in this step. The second step of the model accounts for an irreversible rearrangement of the ELP on the surface where ELP is no longer able to be rinsed off (Eq. 2). Our previous work has shown this model to describe the adsorption of other peptides to a gold surface^55^. We note here that peptides could still transition under stimuli even when the rearrangement step is irreversible.

1 $${\text{ELP + Au }}\underset{{{\text{k}}_{{\text{d}}} }}{\overset{{{\text{k}}_{{\text{a}}} }}{\longleftrightarrow}}{\text{ELP}}_{{\text{i}}} {\text{Au}}$$

2 $${\text{ELP}}_{{\text{i}}} {\text{Au}} \mathop{\longrightarrow}\limits^{{{\text{k}}_{{\text{t}}} }}{\text{ELP}}_{{\text{r}}} {\text{Au}}$$

Au in Eqs. 1 and 2 represents an adsorption site for the reversibly adsorbed ELP at initial adsorption (ELP_i_). k_a_ and k_d_ are rate constants of the association and dissociation of ELP on the Au site, respectively. The second reaction involves irreversible reorientation of the adsorbed ELP during the grafting, where k_t_ represents the rate of the rearrangement and ELP_r_Au is the rearranged ELP immobilized on the Au site. All kinetic fits are shown in Fig. S5.

**Figure 3:**
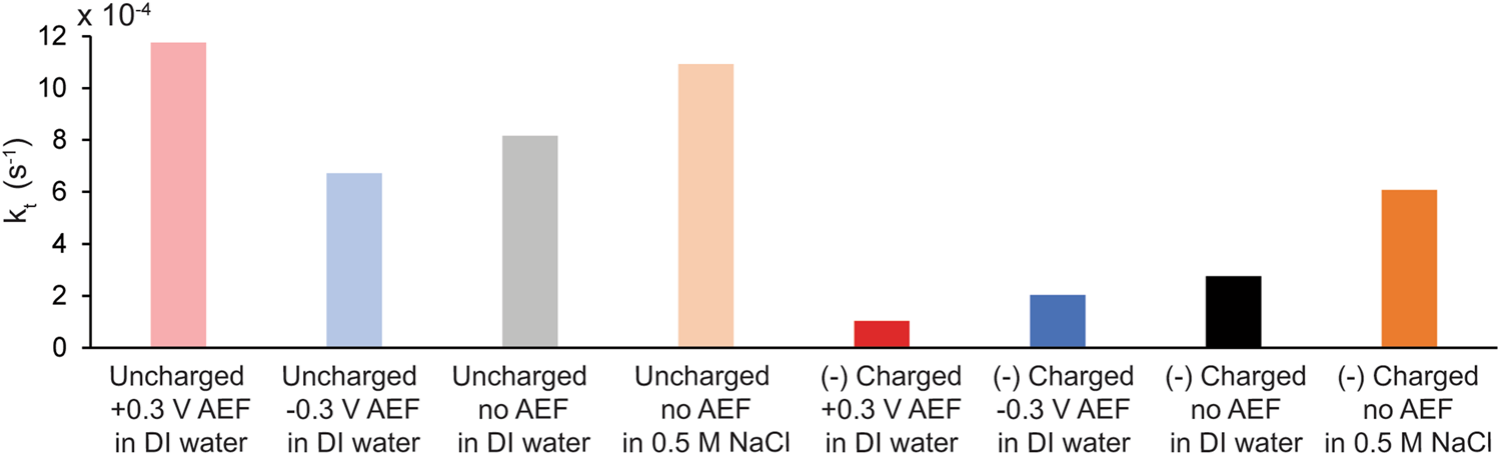
The charge of the ELP has an impact on the rate of rearrangement (k_t_) as peptides adsorb to a gold surface. Values of k_t_ were obtained via nonlinear curve fitting of time-resolved frequency monitoring data (found in Fig. 2) with a bi-exponential kinetic model described in Eq. 1 and 2. Standard errors of the k_t_ parameter estimates were 0.04–0.25 × 10^−4^ s^−1^ (error bars not shown due to small size). Note that the y-axis should be multiplied by 10^−4^.

Fitting the bi-exponential adsorption and rearrangement model to frequency shift with time yielded kinetic parameters k_a_, k_d_ (Fig. S6) and k_t_ (Fig. 3) for the peptide adsorption on gold QCM sensors (Fig. 2). The parameters k_a_ and k_d_ have been well described in the literature and can be used to calculate ∆G_ads_ for the thiol-gold interaction (Eq. 3), where R is the gas constant and T is the temperature^56^. The ∆G_ads_ seen in this work was − 7.69 ± 0.12 and − 7.78 ± 0.16 kcal/mol for charged and uncharged ELP respectively, typical of thiol-gold interactions^55,57–59^ and indicative of a spontaneous process. There was minimal impact of the AEF on ∆G_ads_ under the testing conditions of this work as seen in Fig. S7.

3 $$\Delta {\text{G}}_{{{\text{ads}}}} {\text{ = - RTln(}}\frac{{{\text{k}}_{{\text{a}}} }}{{{\text{k}}_{{\text{d}}} }})$$

The charge of the ELP had an impact on the k_t_ values, however. A higher k_t_ was observed for uncharged ELP regardless of AEF, indicating this peptide undergoes the reorientation process more quickly than the (−)charged ELP. We speculate this difference is likely due to the more hydrophobic nature of uncharged ELP, leading to more hydrophobic interactions between and/or within peptide chains that propagate the reorientation. We speculate that the lower rearrangement in (−)charged ELP could be due to the lower hydrophobicity, charge repulsion, or potentially the association of the carboxylic acid moiety interacting with the gold preventing rearrangement. The AEF had minimal impact on the values of k_t_, suggesting that the reorientation process is dependent only on the properties of the peptide adsorbed to the surface.

To further explore the role of charge in the rearrangement kinetics, we compared the k_t_ measured for (−)charged ELP grafted under no AEF and no NaCl to that grafted under no AEF in 0.5 M NaCl. The k_t_ nearly doubled when (−)charged ELP was grafted in salt, and was close to the k_t_ of uncharged ELP in water. The charge shielding with NaCl seems to increase the rate of rearrangement. For uncharged ELP, grafting with and without NaCl under no AEF yielded similar k_t_ values. Overall, the presence of a negative charge in the ELP had an impact on the rearrangement kinetics. To further probe the grafting mechanisms and understand how the negative charge and AEF impacted the viscoelastic properties as the peptides adsorbed, dissipation versus frequency (DF) plots were investigated.

### Study of grafting mechanism via DF plots

**Figure 4:**
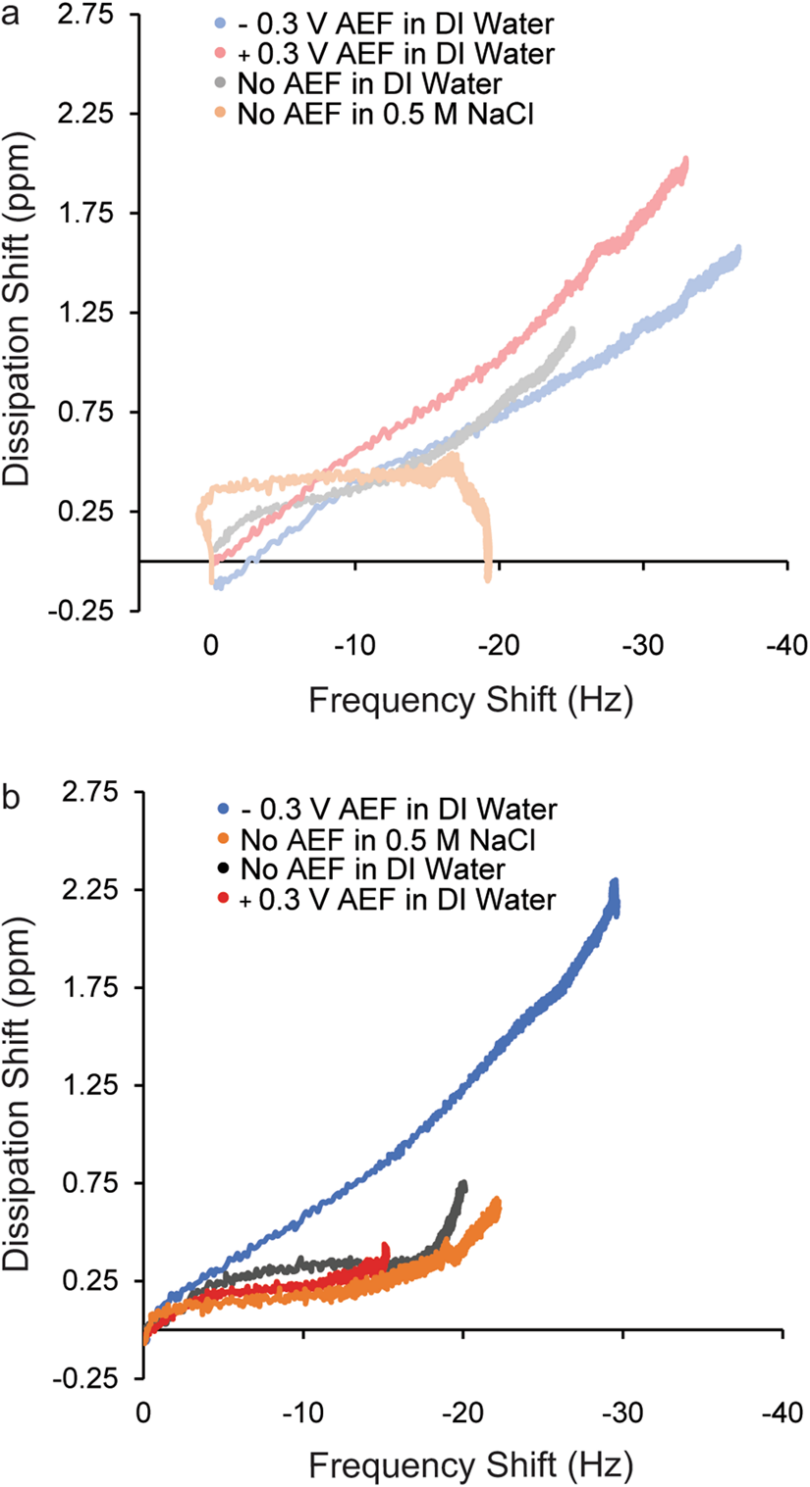
DF plots reveal that peptide charge, salt and AEF influence grafting mechanism. Data are shown from the grafting of (**a**) uncharged ELP (light color) and (**b**) (−)charged ELP (bold color) at different grafting conditions: − 0.3 V AEF in DI water (light blue and blue, respectively), + 0.3 V AEF in DI water (pink and red, respectively), no AEF in DI water (gray and black respectively), and no AEF in 0.5 M NaCl (light orange and orange respectively). All voltages are vs. a Ag/AgCl reference.

Grafting mechanism has been known to impact the final morphology of similar thermoresponsive polymers^46,60^. Therefore, DF plots were examined for all grafting conditions for uncharged (Fig. 4a) and (−)charged (Fig. 4b) ELP in this study. In these plots an increase in dissipation represents a film becoming more viscoelastic and a decreases in dissipation indicates a film becoming more rigid as mass accumulates on the sensor. Generally, the DF plots in this study were characterized by two distinct phases of adsorption which support the adsorption and rearrangement kinetic model discussed in the previous section. In the first phase, peptides initially adsorbed with increasing dissipation, but then the DF curves began to flatten indicating less change in viscoelasticity as peptides populated the surface of the gold. Then in phase two, another increase in viscoelasticity was observed as peptides rearranged. The point at which the second phase began appeared to be highly correlated with k_t_, where samples with low k_t_ were observed to have increases in viscoelasticity later in the overall adsorption process (Fig. 4b, (−)charged peptide grafted with no AEF and at + 0.3 V and − 0.3 V vs. Ag/AgCl in DI water).

The first stage of the adsorption depended on peptide charge, the presence of salt, and on AEF. Uncharged ELP grafted in DI water had similar DF profiles regardless of AEF with a sharper increase in viscoelasticity/dissipation shift and then a slower increase (Fig. 4a). In contrast, the adsorption profile of the (−)charged ELP grafted without an AEF and at + 0.3 V vs. Ag/AgCl AEF had a sharp initial increase in viscoelasticity followed by flat DF profile. The flatter DF curve indicates that the peptides lying flatter against the gold surface. In addition, electric field had an impact on the adsorption mechanism of the (−)charged ELP assembled at − 0.3 V vs. Ag/AgCl AEF, which was characterized by a relatively continuous increase in viscoelasticity as the peptide absorbed. The higher viscoelasticity could indicate that the (−)charged C-termini were oriented upward away from the gold surface, drawing more water to the surface-bound ELP layer.

When peptides were grafted in no AEF in 0.5 M NaCl, it was observed that the first phase of the adsorption was characterized by a flat DF profile after a short initial viscoelastic increase. This suggests that the peptides more readily collapse into a compact state in the salt solution. In the second stage of adsorption where rearrangement occurs, we observe another change in slope, occurring around the same time in both peptides. In the grafting of the charged peptide, the rearrangement appeared to start sooner in the process than the samples assembled in DI water, further corroborating our kinetic modeling results suggesting that shielding the negative charge led to faster rearrangement (higher k_t_). We also note that as the uncharged peptide assembled in NaCl, the second stage was characterized by a slow increase in viscoelasticity, but then a sudden drop. Speculatively, the ELP may have undergone transition as it assembled in the salt, driving away water molecules from the peptide layer on gold.

Overall, the DF plots support an adsorption and rearrangement mechanism for the elastin peptides as they are grafted to the surface and that peptide charge, the presence of salt, and AEF can influence the peptide grafting mechanism. Next, AFM was utilized to understand how these differences in mechanism impact the morphology of assembled ELP.

### Morphological investigation of grafted ELP using AFM images

**Figure 5:**
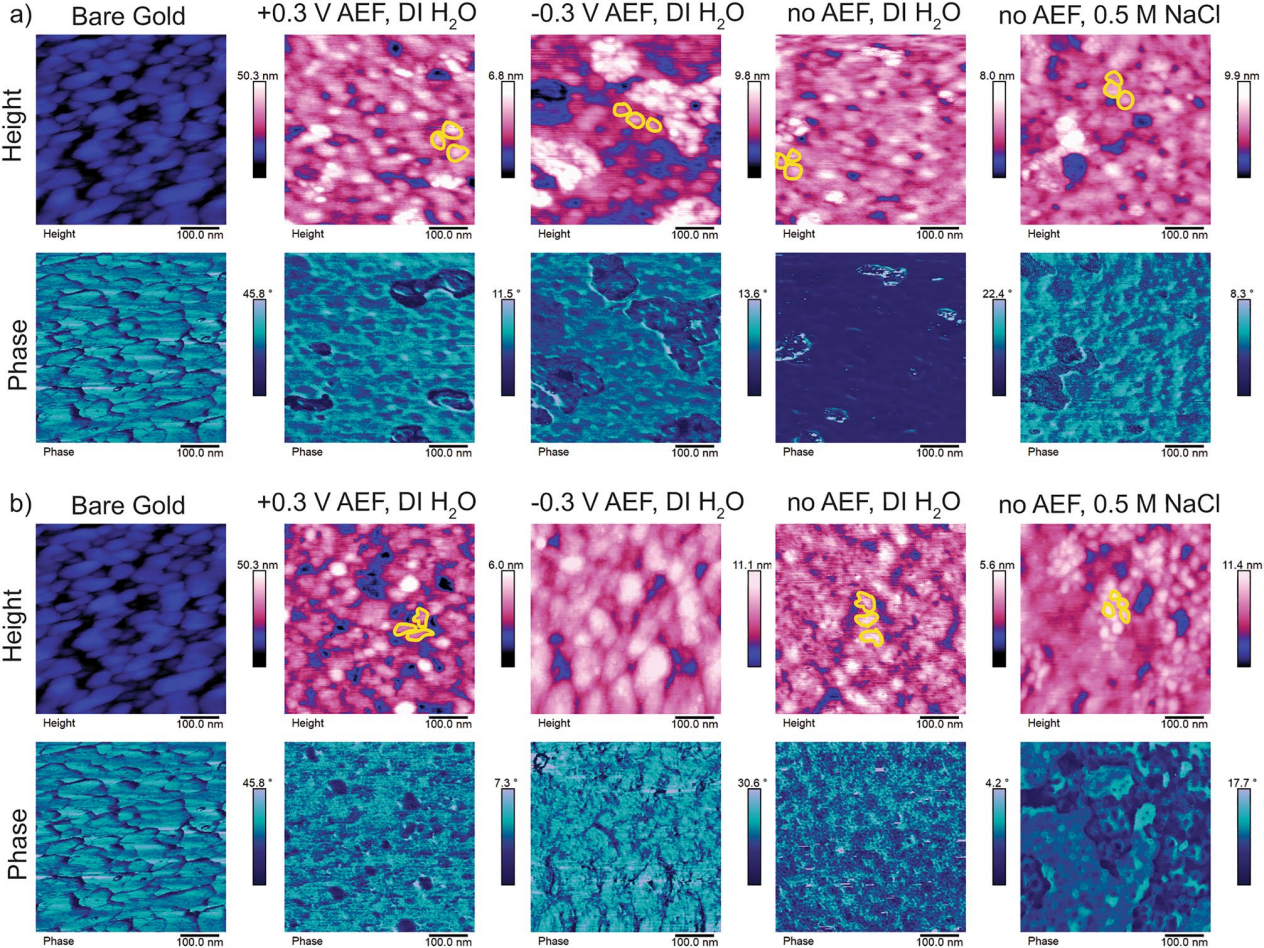
AFM images of grafted ELP show that charge/hydrophobicity and AEF impact morphology. Height and phase contrast images of (**a**) uncharged ELP and (**b**) (−)charged ELP grafted on gold QCM sensors at + 0.3 V AEF in DI water, − 0.3 V AEF, with no AEF in DI water, and with no AEF in 0.5 M NaCl. A bare gold QCM sensor is provided for reference. Example morphologies are outlined in yellow. All voltages are vs. a Ag/AgCl reference.

Observable differences in surface-bound ELP morphology grafted at different conditions were visualized using AFM. Figure 5 shows topography and phase contrast images for uncharged (Fig. 5a) and (−)charged ELP (Fig. 5b) grafted at +0.3 V vs. Ag/AgCl in DI water, at − 0.3 V vs. Ag/AgCl in DI water, with no AEF in DI water, and with no AEF in 0.5 M NaCl. To quantify the observed morphologies in the AFM images correlation length (ξ) and the radius of gyration (R_g_) were estimated. ξ is the distance between the centers of two separate ELP blobs and R_g_ is he radius of an ELP blob. Examples of ELP blobs are outlined in yellow in Fig. 5. The morphology of the grafted ELP was considered to be brushes when blobs were not observable, mushrooms when ξ > 2R_g_^61^, and pancakes when blob morphology was irregular, and with lower topography than mushroom. AFM revealed that uncharged ELP generally formed mushroom-like morphologies, with R_g_ ~ 10–25 nm and ξ ~ 50–100 nm. The (−)charged ELP formed flatter pancake-like morphologies, however, when grafted in the presence of salt which shields the charge, mushroom-like morphologies were observed similar to uncharged peptide. Interestingly, when the (−)charged ELP is grafted at − 0.3 V vs. Ag/AgCl in DI water, blobs were not readily observed, indicating a brush-like morphology, and supporting the DF plots suggesting a more upright (normal to the surface) configuration of the molecules. Generally, the phase images support the topography images and presence of three distinct morphologies of the grafted ELP. To further confirm the morphological differences, surface roughness of the samples was characterized by water droplet contact angle measurement (Fig. S8) which corroborated the findings in AFM images as discussed in the SI.

Collectively, our results indicate that the morphology of ELP grafted to gold was impacted by the hydrophobicity and/or presence of charge in the peptide, which was correlated with a higher rearrangement kinetic constant. Specifically, peptides with higher hydrophobicity and/or no charge rearranged faster and that rearrangement may have played a role in the observed morphology. Therefore, while peptides may have laid flat on the surface in the beginning stage of the adsorption process, such as the (−)charged peptide grafted in salt, with a shielded negative charge and higher rearrangement constant the peptide was capable of forming a mushroom morphology in the timeframes of this study. This highlights the importance of kinetics in the grafting process. In addition, an electric field judiciously used on charged peptides impacts the grafted orientation such that even with a lower rearrangement constant, such as in (−)charged peptide grafted in the − 0.3 V vs. Ag/AgCl AEF condition, the peptides assume a more upright position normal to the surface resulting in higher loading, higher viscoelastic properties and ultimately a brush-like morphology. Table 2 summarizes the collective results from this study and Fig. S9 shows a schematic summary of the ELP adsorption mechanisms elucidated by our study. Next, the importance of morphology on surface-bound transition behavior was investigated.

**Table 2:** The morphology of ELP grafted on gold is impacted by hydrophobicity/charge, rearrangement kinetics and AEF. The conditions of peptide grafting experiments are described (peptide type, assembly voltage, and presence of salt) along with their impact on the rearrangement constant (k_t_) and grafted ELP morphology.

Peptide	Assembly voltage versus Ag/AgCl (V)	Salt (M)	Rearrangement constant, k_t_ (s^−1^)	Morphology
Uncharged ELP	0.3	0	0.0012	Mushroom
Uncharged ELP	− 0.3	0	0.00067	Mushroom
Uncharged ELP	No AEF	0	0.00082	Mushroom
Uncharged ELP	No AEF	0.5	0.0011	Mushroom
(−)Charged ELP	0.3	0	0.00010	Pancake
(−)Charged ELP	− 0.3	0	0.00020	Brush
(−)Charged ELP	No AEF	0	0.00028	Pancake
(−)Charged ELP	No AEF	0.5	0.00061	Mushroom

### The impact of electric field assembly on surface-bound transition behavior

**Figure 6:**
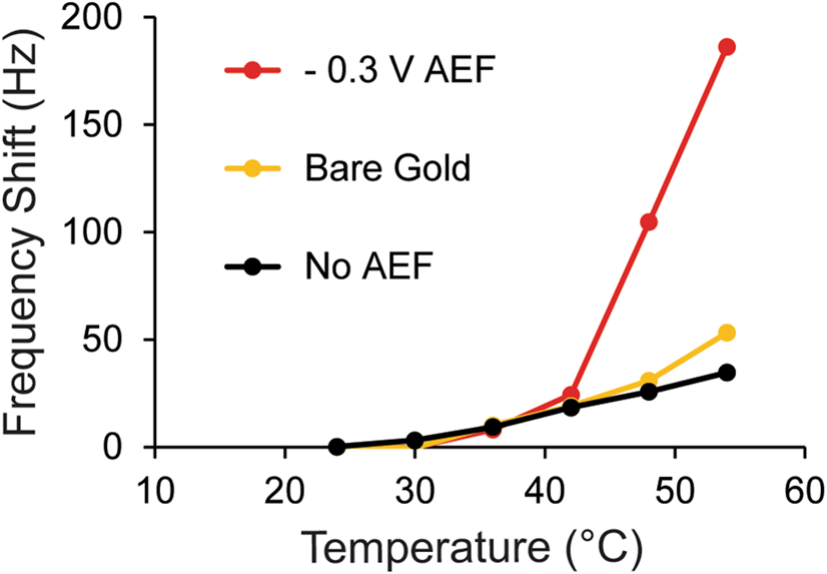
The surface-bound transition behavior of (−)charged ELP is impacted by assembly in an AEF. A bare gold control (yellow), (−)charged ELP assembled without an AEF (black) and (−)charged ELP assembled in − 0.3 V AEF vs. Ag/AgCl (red) were heated in 6 °C increments while the frequency shift was monitored. Lines between data points are added to help readers more clearly see the frequency trends.

To assess the impact of electric field assembly on surface-bound transition behavior, (−)charged peptides were grafted to gold using an electrochemical QCM-D module in DI water with and without an AEF at − 0.3 V vs. Ag/AgCl, as described for results displayed in Fig. 2. (−)Charged ELP was chosen for this demonstration because it responded the most to electric field. Furthermore, ELPs designed for sensing applications are likely to contain negatively charged DNA or charged peptide aptamers^62^. Following assembly, the temperature was increased in increments of 6 °C from 24 °C to 54 °C while monitoring the frequency shift. Averages of frequency were taken at each temperature and plotted in Fig. 6 for (−)charged ELP assembled in − 0.3 V vs. Ag/AgCl AEF, assembled without an AEF, and for a bare gold control. When the (−)charged ELP grafted without an AEF was heated, there was a linear relationship between temperature and frequency shift, similar to the bare gold control. This is indicative of behavior where there is no transition^46^. The result could mean that the orientation of the peptide, likely laying flatter against the surface, results in a transition temperature out of the tested range. However, when the same peptide was grafted in − 0.3 V vs. Ag/AgCl AEF, the slope of temperature versus frequency increased starting at 42 °C, which is indicative of phase transition behavior^46^. Fig. S10 shows both heating (to 60 °C) and cooling curves of two samples of (−)charged ELP assembled at − 0.3 V vs. Ag/AgCl AEF. Reversible behavior was observed in both samples and was repeatable, with some hysteresis. The observed hysteresis will be investigated in the future to determine potential causes. These data demonstrate the importance of surface-grafted ELP orientation on the transition behavior measured using QCM-D temperature ramping.

## Conclusions

This article presented a study of the surface-grafting of short ELP peptides onto a gold surface, and specifically investigated how an electric field and peptide charge impacted the ELP hydrated mass loading, grafting kinetics, final morphology and structural transition properties. Two peptides were designed, both containing a thiol moiety for gold-binding, five repeats of elastin with valine as the guest residue, and a FRET pair. One peptide had a negative charge on the C-terminus, whereas the other peptide was uncharged. Temperature dependent DLS and fluorescent quenching experiments revealed that both designed peptides were capable of reversible transition behavior. Time-course QCM-D data and ellipsometry measurements revealed that when assembled at − 0.3 vs. Ag/AgCl AEF in our system, the hydrated mass loading of the (−)charged peptide increased compared to loading without AEF and loading of peptides grafted at + 0.3 V AEF, indicating that a sufficiently high AEF impacted the orientation of the charged peptide by pushing the charged group away from the surface. Investigation of dissipation versus frequency plots revealed that (−)charged peptide assembled at − 0.3 V vs. Ag/AgCl had higher viscoelastic properties throughout assembly compared to assembly in other conditions, providing further evidence of a more upright orientation. Dissipation versus frequency plots and time-course QCM-D kinetic modeling of frequency shifts revealed that ELP peptides assembled via a two-step adsorption and rearrangement process. Notably, we discovered that rearrangement kinetic constants were highly correlated with the final morphology of the grafted ELP, with fast rearrangement correlated with mushroom-like morphologies, and slower rearrangement correlated with flatter pancake-like morphologies. Charged peptides had slower rearrangement kinetics, but by shielding the charge with salt during assembly, it was discovered that the rearrangement constant could be increased, indicating that hydrophobicity/charge were significant drivers in the rearrangement process. In addition, assembly of the (−)charged peptide at − 0.3 V versus Ag/AgCl AEF resulted in brush-like morphologies, further corroborating that the AEF had an impact on grafted ELP orientation. Moreover, orientation controlled by AEF impacted the transition behavior of surface-bound (−)charged ELP measured using QCM-D temperature ramping. Collectively, these results highlight that judicious use of peptide design via charge/hydrophobicity, kinetic assembly and AEF can control the grafted morphology of ELP where the grafted morphology has an impact on properties such as transition behavior. Overall, this study provides a means for achieving desirable and predictable surface properties pivotal to many surface-grafted ELP applications (e.g., sensing, tissue engineering, capture/release application).

## Methods

### Peptide synthesis

The elastin peptides were purchased from GenScript in the form of dehydrated powder with > 95.0% purity. Purity was confirmed by GenScript via high-performance liquid chromatography and certificates of analysis were sent with each peptide. Fig. S11 shows the chemical structure of (−)charged ELP.

### Dynamic light scattering (DLS)

The hydrodynamic radii of the peptides in DI water were measured with a Möbiuζ (Wyatt Technology Corporation) dynamic light scatterer, under controlled temperature. For temperature ramping measurements, the ramping rate was 2 °C/min from 20 °C to 60 or 70 °C and each measurement of hydrodynamic radius was the average of three acquisitions. A quartz cuvette with a 10 mm pathlength was used. Data collection and analysis were carried out using the DYNAMICS software package (Wyatt Technology Corporation).

### Fluorescence measurement

The fluorescence emission of the peptides suspended in aqueous solutions was monitored as the temperature was raised and lowered between 20 °C and 45 °C using a SpectraMax M2 The samples were prepared in 96-well plates at different NaCl concentrations (0.01, 0.05, 0.10, 0.50, 1.00, 1.45 M). The wavelength of excitation was 320 nm and the wavelength of emission was 420 nm. Each measurement was the average of three acquisitions. Data from blank solutions (aqueous solution with the same set of NaCl concentrations) were subtracted from the data gathered on samples.

### Quartz crystal microbalance with dissipation (QCM-D)

To study the grafting of elastin peptides on a gold surface, peptides were flowed past a gold-coated quartz sensor in a temperature-controlled electrochemical quartz crystal microbalance with dissipation monitoring (EQCM-D) module from Biolin Scientific (QEM 401). Each peptide was dissolved in DI water or 0.5 M NaCl aqueous solution at a concentration of 10 µg/mL. The temperature of the EQCM-D module was set at 20 °C throughout all grafting conditions. The peptide solution was pumped into the EQCM-D module at a constant flow rate of 0.150 mL/min once the frequency and dissipation signals had stabilized after a few hours of baseline with the background solvent. Electric field, as appropriate, was applied at − 0.3 V and + 0.3 V vs. Ag/AgCl (3M KCl) with a PGSTAT101 Metrohm potentiostat. The module houses a Pt counter electrode plate that is 0.8 mm away from the gold working electrode. The hydrated mass uptake of the peptide layers grafted onto the gold surface was calculated from the recorded frequency by the QSense software as we have described previously using the Sauerbrey model^55^. The 3rd overtone (n) of the frequency and dissipation signals was selected for mass calculation, kinetics, and the DF analyses due to its stability and the noise-free nature of the data.

### Fourier-transform infrared (FTIR) spectrometry

Peptide films were characterized with a Nicolet iS50 spectrometer with a VeeMAX-II specular reflectance accessory. The accessory was equipped with a wire grid ZnSe polarizer set at 90°. The grazing angle of incidence was set at 65° for optimum FTIR signal. The FTIR spectra were acquired with a mercury-cadmium-tellurium (MCT) detector with resolution of 4 cm^−1^ and 600 scans for each sample. The accessory was purged with nitrogen gas and the MCT detector was cooled with liquid nitrogen prior to the FTIR measurements. Background spectra of a bare gold QCM sensor were taken and subtracted from those of the peptide films.

### Spectroscopic ellipsometry

The dried thickness of each grafted peptide film on the gold QCM sensor was measured in air, using a Horiba Uvisel DUV-NIR spectroscopic ellipsometer. Data were acquired at a 70° angle of incidence over an energy range of 1—5 eV (248—1240 nm). Ellipsometry data were analyzed using Delta Psi software; the model consisted of a Au substrate layer topped with a Cauchy transparent layer. Based on the work led by Mutafa^63^, the parameters of the Cauchy layer were set to A = 1.792, B = 0.1, C = 0, and the model was fit over the energy range 1.2–3.4 eV. The reported thickness of each dried peptide film is the average of two measurements reflecting two different spots on the same layer. Chi squared values for the fits were < 1 for all values reported.

### Kinetic modeling

Curve fits to the reaction scheme shown in Eqs. 1 and 2 were conducted for QCM-D adsorption data using the third overtone in Python. The full biexponential fitting equation can be found in our previous work^55^. Specifically, the lmfit package was utilized to conduct nonlinear least squares fitting while reporting fitting parameters and model errors.

### Atomic force microscopy (AFM)

The morphology of each peptide film was assessed with Bruker Dimension 3100 Veeco Digital Instruments AFM. Tapping mode with a FESPA-V2 AFM probe (tip specifications: antimony doped Si, nominal radius of 8 nm; probe specifications: resonant frequency of 75 kHz, nominal spring constant of 2.8 N/m) was used. The scans were taken at 500 × 500 nm^2^ at a scan rate of 0.256 Hz. The AFM images were processed and analyzed with NanoScope Analysis V 1.50 software. The peptide film samples on QCM sensors were air dried and the AFM scans were taken in a chamber at room temperature and under atmospheric pressure. Semi-quantitative analysis of correlation lengths and the radius of grafted peptides were carried out using ImageJ software.

### Contact angle measurement

Water contact angles were measured using a First Ten Angstroms (Newark, CA) FTA200 system. Two 5-µL water droplets were pipetted one at a time onto each peptide film grafted on a QCM sensor. Each water droplet was allowed to equilibrate for 30 s prior to photographing and contact angle measurements. Photos and measurements were taken using Fta32 Video 2.0 software. Left and right angles were averaged for each droplet to obtain two measurements per sensor sample.

## Characterization of grafted ELP transition behavior using QCM-D temperature ramping

To study the grafted (−)charged ELP transition behavior using QCM-D temperature ramping, each peptide was grafted on a gold-coated quartz sensor in a programmed temperature-controlled electrochemical quartz crystal microbalance with dissipation monitoring (EQCM-D) module from Biolin Scientific (QEM 401). 10 µg/ml of each peptide solution was prepared in DI water. The flow rate of peptide solution speed was 0.025 mL/min. The temperature ramping program started after the peptide was assembled (i.e., when frequency and dissipation signals had stabilized and after DI water rinsing). The assembly process took 40 min. Samples of peptide assembled with no AEF and assembled in − 0.3 V vs. Ag/AgCl AEF were subjected to temperature ramping. The temperature program of the EQCM-D module was set from 24 to 60 °C with an increment rate of 6 °C. QSense software was utilized to record temperature, dissipation, and frequency with time. The 9th overtone (n) of the frequency was selected for analysis.

### Statistics

All statistical analysis (except non-linear curve fitting, see Kinetic modeling section) was performed using Minitab Version 19.2020.1. A significance level of α = 0.5 was chosen for all statistical tests. Specifically, linear regression was performed in Fig. S2 and single-factor ANOVA was performed on data in Fig. S4 and S8. Tukey’s post-hoc analysis was performed after confirming treatment significantly impacted the response to determine which groups were statistically different/similar. Data are represented by the average ± the standard deviation, unless the figure caption indicates otherwise.

## Supplementary Information

Supplementary Information.

## Data Availability

Data found in the main text of this manuscript can be found on Harvard Dataverse: https://doi.org/10.7910/DVN/TGGCTT.
