# Supplementary material for: Grafting of short elastin-like peptides using an electric field
Source: Sci Rep. 2022 Nov 4;12:18682. doi: 10.1038/s41598-022-21672-9 (PMC9636273; doi:10.1038/s41598-022-21672-9)
Supplement: Supplementary file 1 — Supplementary Information. [file 41598_2022_21672_MOESM1_ESM.docx]

***Supporting Information***

**Title: Grafting of short elastin-like peptides using an electric field**

**Authors:** Nuttanit Pramounmat,^1^ Sogol Asaei,^1,#^ Jacob D. Hostert,^1,#^ Kathleen Young,^2^ Horst von Recum_,_^2^ and Julie N. Renner^1,*^

^1^Department of Chemical and Biomolecular Engineering, Case Western Reserve University

^2^Department of Biomedical Engineering, Case Western Reserve University

^#^Authors contributed equally

^*^Corresponding author

**Transition behavior measured via FRET.** Prior to investigating the impact of electric fields on ELP grafting and subsequent transition behavior, the stimuli-responsiveness of the designed peptides was confirmed in solution. Fluorescent probes can provide insight into the structural transition of elastin-like polypeptides^1,2^. Both structural change and aggregation could cause a quenching of a fluorophore attached to the ELP.

Increasing solution temperature from 20°C to 45°C and NaCl concentration from 0.01 M to 1.45 M and ELP concentration from 0.05 to 5 mg/mL resulted in increased fluorescence quenching, suggesting a structural transition of the uncharged (**Fig. S1a-d)** and negatively charged ELP (**Fig. S1e-h)**, respectively. The observed quenching behavior can be explained by structural changes that occur during transition (from soluble and extended to insoluble and collapsed). The trend of increasing temperature^3^, ELP concentration^4^ and salt^5,6^ corresponding with more quenching, interpreted as a transition to insoluble and collapsed ELP from soluble and extended ELP, agree with literature describing similar trends. In addition, the uncharged (more hydrophobic) peptide quenches more dramatically at lower NaCl concentration, temperature and ELP concentration as compared to the charged (more hydrophilic) peptide, as expected^7,8^. Based on analysis of the heating data shown in **Fig. S1**, we estimated the transition temperature at 0.01 M NaCl and the lowest concentration of 0.05 mg/mL. The estimate was performed by plotting the relative fluorescence of both charged and uncharged ELP as a function of temperature. There is a significant linear relationship between relative fluorescence and temperature for the negatively charged ELP, but a clear deviation from linear behavior between 20-40°C for the uncharged ELP (**Fig. S2**). Therefore, while the negatively charged ELP (more hydrophilic) does not transition in 0.01 M NaCl at 0.05 mg/mL the uncharged ELP (more hydrophobic) has a transition temperature between 20 and 40°C at the same conditions.


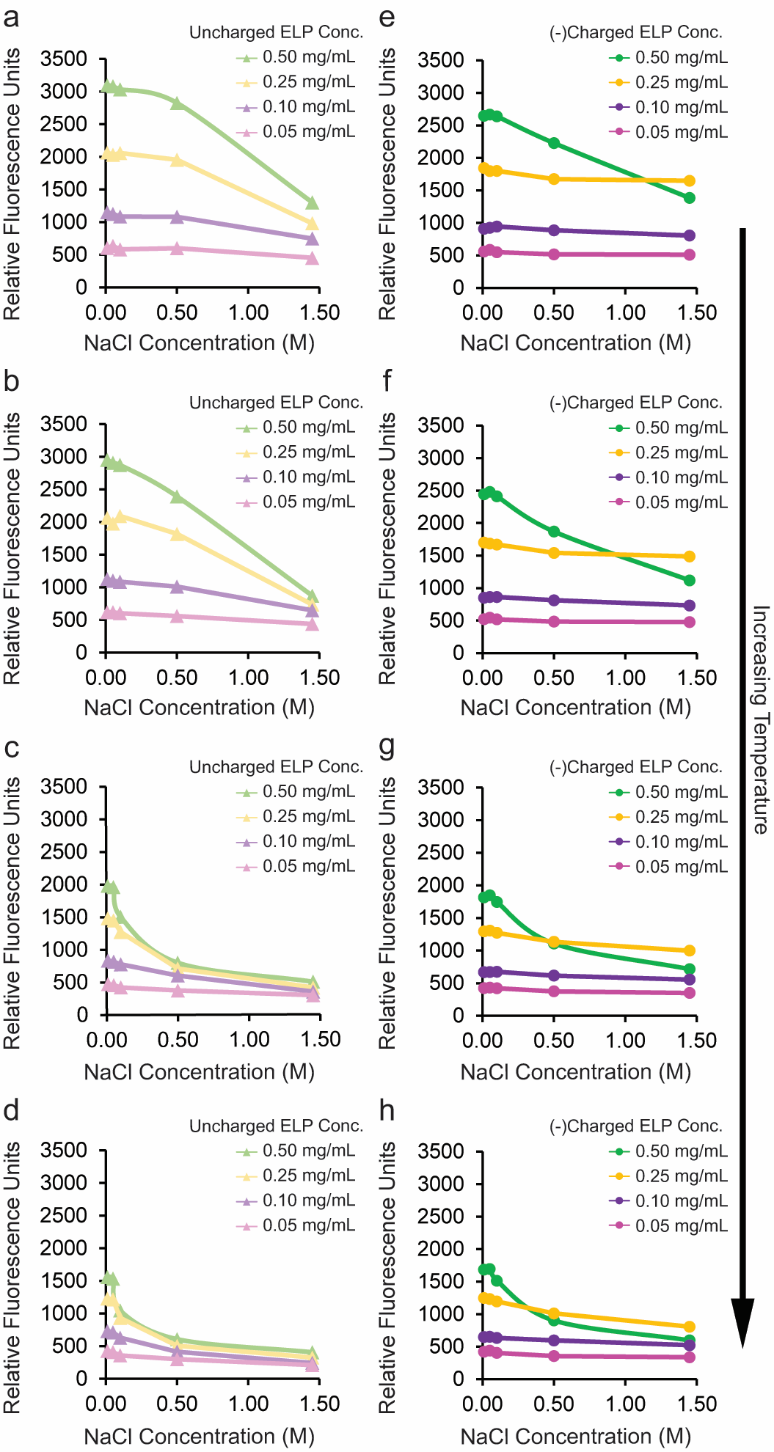


**Fig. S1** Fluorescence measurements taken in a 96-well plate suggest uncharged ELP (a-d, data represented as triangles in light colors) and negatively charged ELP (e-h, data represented as circles in bold colors) undergo conformational change as they respond to a rise in temperature (a,e 20°C; b,f 30°C; c,g 40°C; d,h 50°C) and NaCl concentration (0.01, 0.05, 0.10, 0.50 and 1.45 M). ELP concentration ranges between 0.50 (green), 0.25 (yellow), 0.10 (purple) and 0.05 (pink) mg/mL. Lines between the data points are meant to guide the reader’s eye, and do not represent a fitted model.


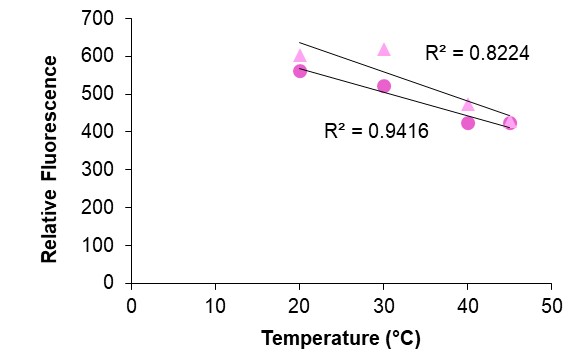


**Fig. S2** Fluorescence measurements for 0.05 mg/mL negatively charged (circles, bold color) and uncharged (triangle, light color) ELP in 0 M NaCl as the temperature is raised from 20°C to 30°C, then 40°C, and then 45°C. Linear regression analysis of variance performed via Minitab indicates the linear relationship between temperature and relative fluorescence in the negatively charged peptide is significant (regression coefficient is significant, p-value < 0.05) whereas the relationship between temperature and relative fluorescence in the uncharged peptide is not significant (regression coefficient is not significant, p-value > 0.05) at α = 0.05.

**Analysis of surface-grafted ELP via Fourier transform infrared (FTIR) spectroscopy.** All samples exhibited two peaks in the amide I region between wavenumbers 1600 cm^-1^ and 1700 cm^-1^ (around 1650-1670 cm^-1^ and 1640-1630 cm^-1^). Peaks in this region are indicative of elastin^9–11^ and multiple peaks in this region have been observed previously^12,13^. Amide II peaks are also observed between 1540-1520 cm^-1^ for all gold-grafted ELP samples. Additionally, a peak around 1400 cm^-1^ is observed only in the negatively charged ELP sample assembled at -0.3 V, which may suggest more free carboxylic acid^14^, corroborating our results suggesting differences in the molecular orientation of the assembled peptides. More free carboxylic acids would be expected in an upright configuration.


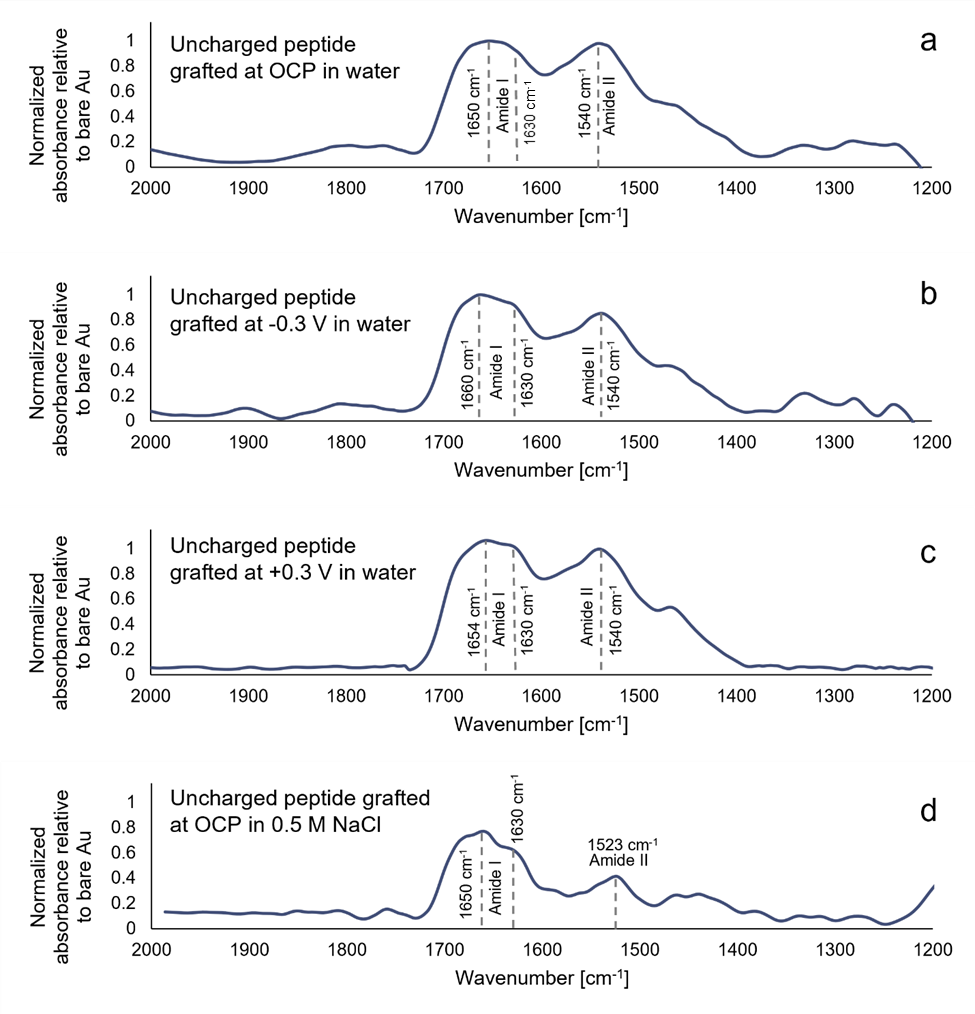


Fig. S3-I FTIR spectra of uncharged ELP grafted at (A) no AEF, (B) -0.3 V AEF, (C) +0.3 V in DI water, and (D) no AEF in 0.5 M NaCl. Typical ELP amide I and amide II peaks are shown.


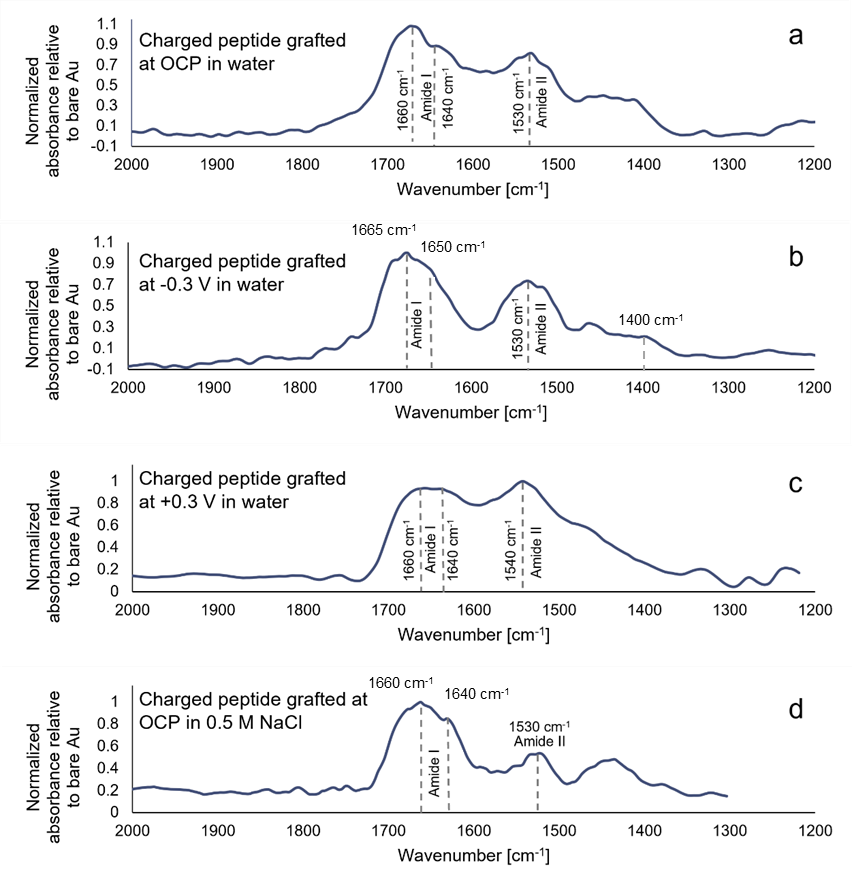


Fig. S3-II FTIR spectra of negatively charged ELP grafted at (A) no AEF, (B) -0.3 V AEF, (C) +0.3 V in DI water, and (D) no AEF in 0.5 M NaCl. Typical ELP amide I and amide II peaks are shown, as well as a peak at 1400 cm^-1^ corresponding to free carboxylic acid groups.

**Analysis of dried surface-grafted ELP via spectroscopic ellipsometry**. Negatively charged ELP grafted at AEF +0.3 V and without AEF in DI water showed lower dried film thicknesses (2.1 and 2.4 nm, respectively), than the rest of the samples (2.9-3.3 nm) and had their own statistical grouping via Tukey’s post-hoc analysis, which provides further support that the molecular orientation of the peptides was impacted by a sufficiently high AEF. Thicknesses trends agreed with the hydrated mass loading measured by quartz crystal microbalance with dissipation and provided further support that the molecular orientation of the peptides was impacted by the AEF.


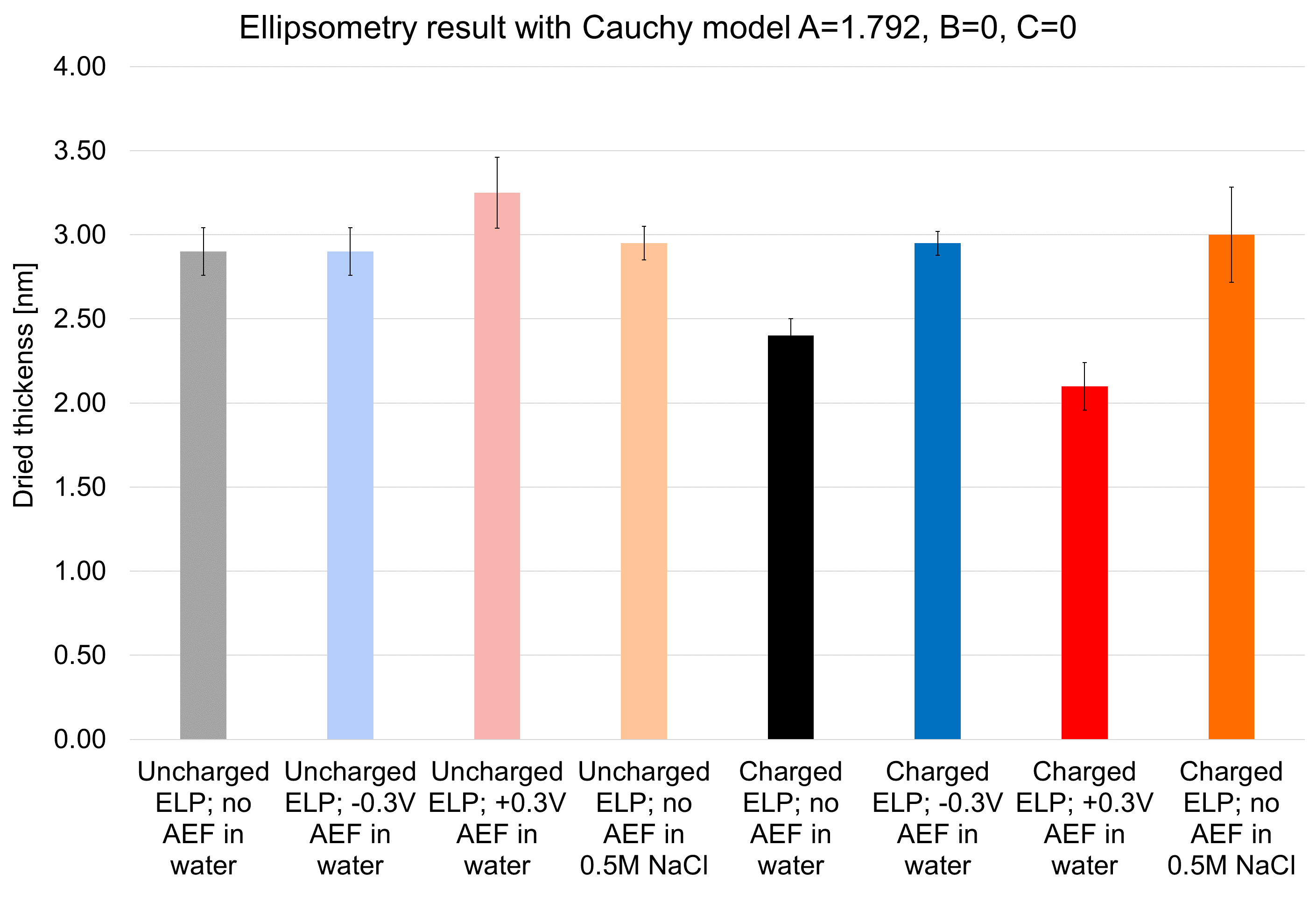


Fig. S4 Dried thicknesses of ELP grafted on gold QCM sensors at different conditions measured via ellipsometry. Bars represent the average of n=2 points on the same sample and error bars represent ± standard deviation.


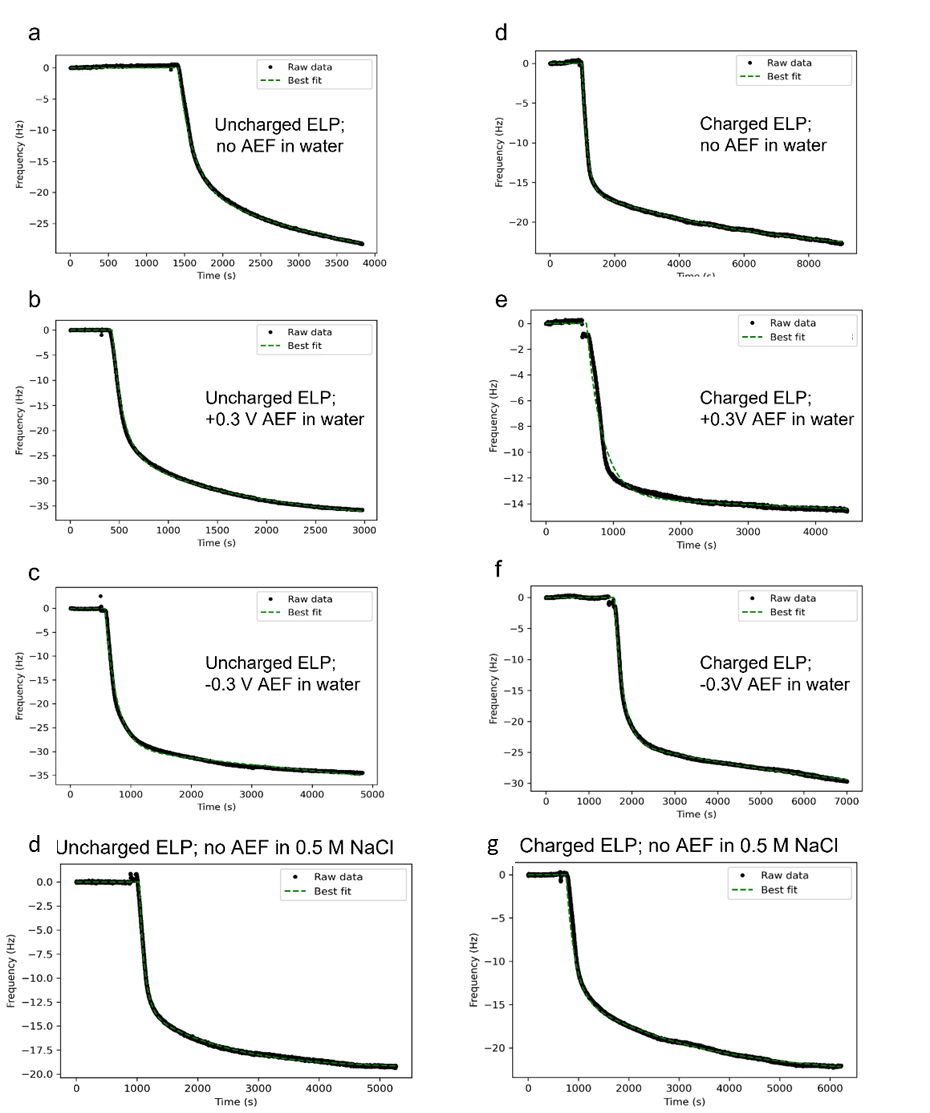


Fig. S5 Kinetic fits for the frequency shifts monitored during the different adsorption conditions: uncharged ELP (a) with no AEF in DI water, (b) at +0.3 V, (c) at -0.3 V, and (d) no AEF in 0.5 M NaCl; negatively charged ELP (a) with no AEF in DI water, (b) at +0.3 V, (c) at -0.3 V, and (d) no AEF in 0.5 M NaCl. Black dots represent data gathered from the quartz crystal microbalance with dissipation, and green dotted lines represent results of nonlinear least-squares curve fitting performed using Python.


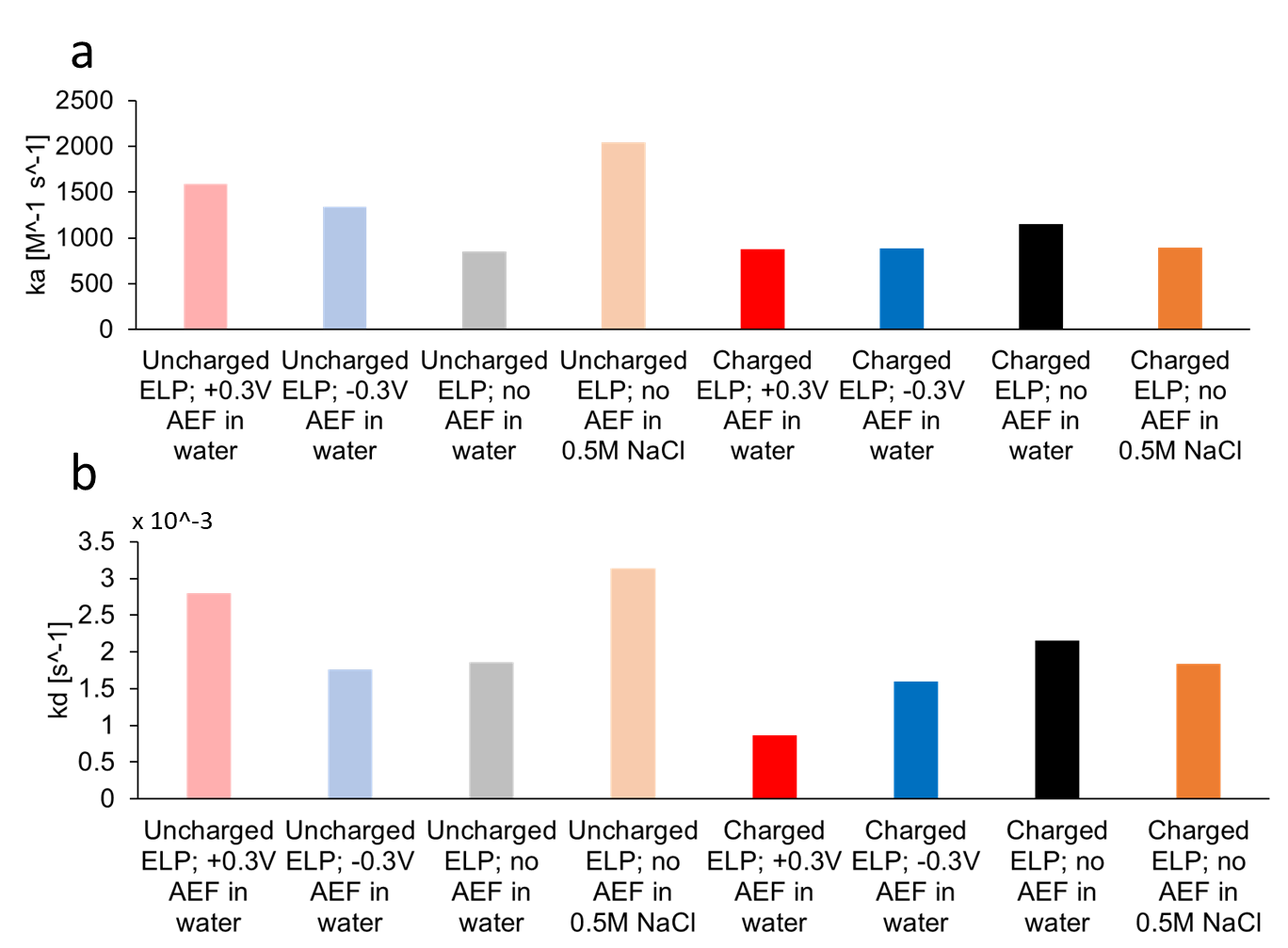


Fig. S6 Values of (a) k_a_ and (b) k_d_ that were obtained via non-linear curve fitting of time-resolved frequency monitoring data (found in Fig. 2) with a bi-exponential kinetic model described in Equations 1 and 2. Note that the y-axis in part b should be multiplied by 10^-3^.


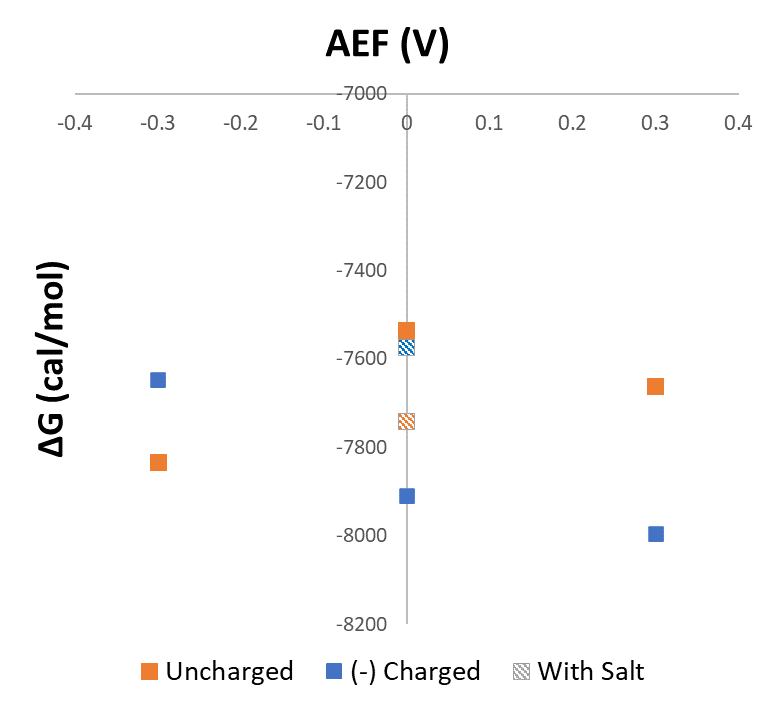


Fig. S7 Values of ∆G_ads_ calculated using Equation 3 in the main text. Uncharged peptide data is represented by orange squares, and negatively charged peptide data is represented by blue squares. Adsorption performed with 0.5 M NaCl are represented by patterned squares.

**Analysis of surface-grafted ELP via contact angle**. The charged ELP grafted at -0.3 V had a statistically similar contact angle as bare gold (a smooth surface which has a higher contact angle), indicating a uniform coverage on the gold surface. Generally, mushrooms and pancake morphologies resulted in relatively low contact angles (a rougher surface which has lower contact angle), corroborating the findings in the AFM images.


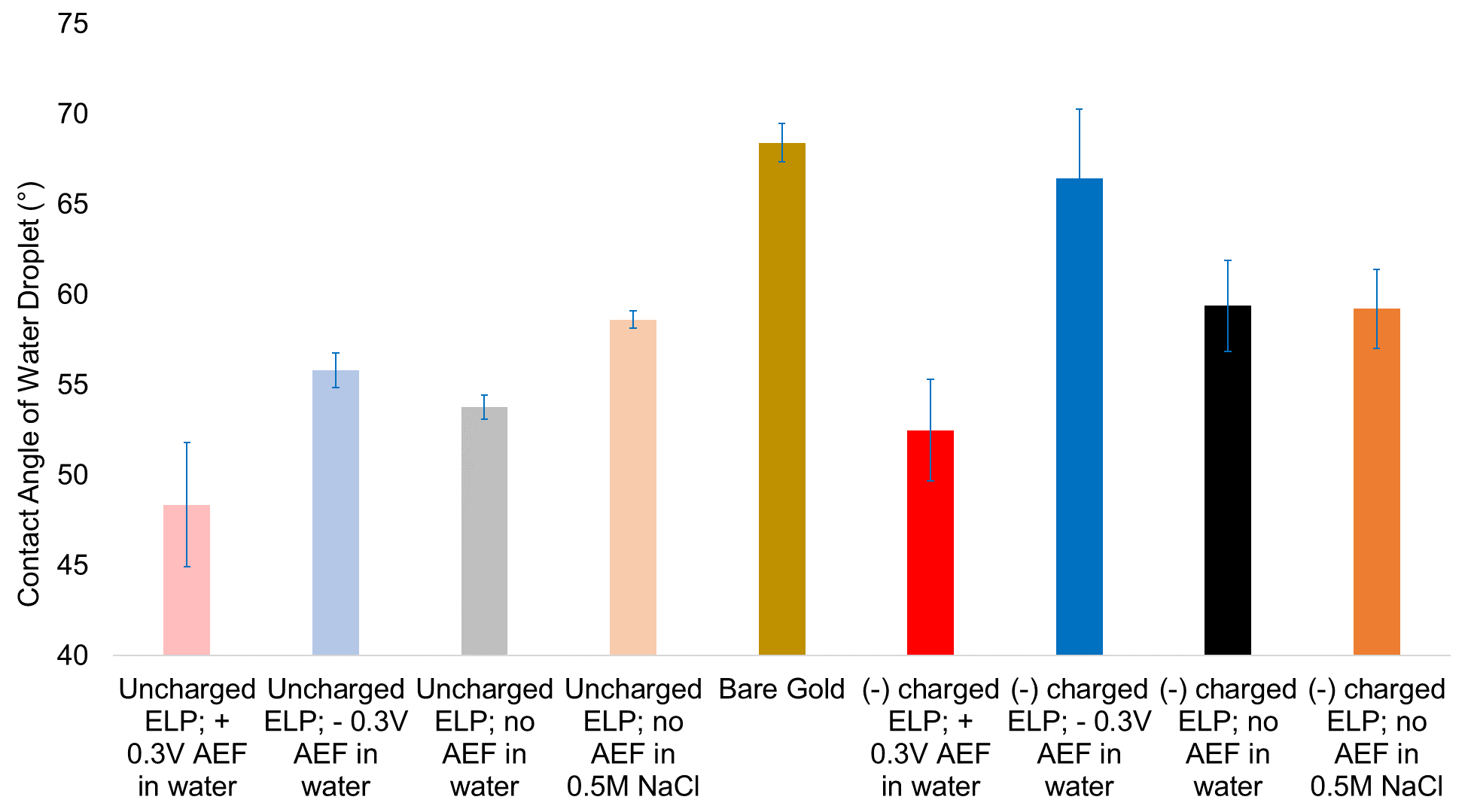


Fig. S8 Contact angles of water droplets on ELP grafted gold QCM sensors at different conditions as labeled on the X axis of the bar graph. Bars represent the average of n=2 droplets on the same sample and error bars represent ± standard deviation.


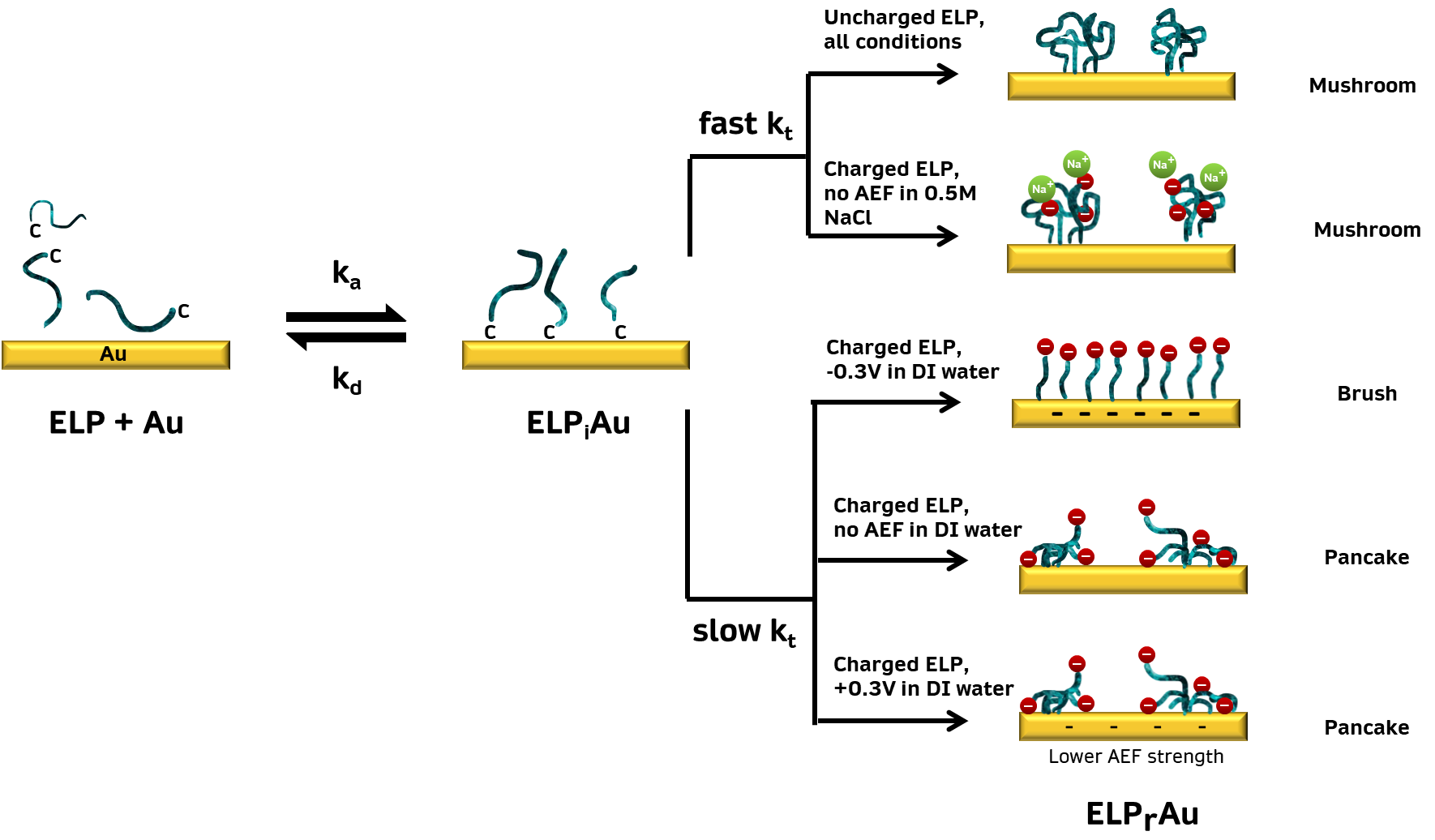


Fig. S9 Summary of the ELP adsorption mechanisms elucidated by our study. The overall adsorption mechanism consists of a reversible attachment followed by an irreversible rearrangement (see Equations 1 and 2 in the main text). k_a_ and k_d_ are rate constants of the association and dissociation of ELP on gold (Au) respectively, k_t_ represents the rate of the rearrangement, and ELP_r_Au is the rearranged ELP immobilized on Au. Blue lines represent ELP, C represents a cysteine amino acid for gold binding, red circles represent a negatively charged peptide terminus and green circles represent the presence of salt. All voltages are vs. Ag/AgCl, and +0.3 V represents a weaker applied electric field in the same direction as -0.3 V.


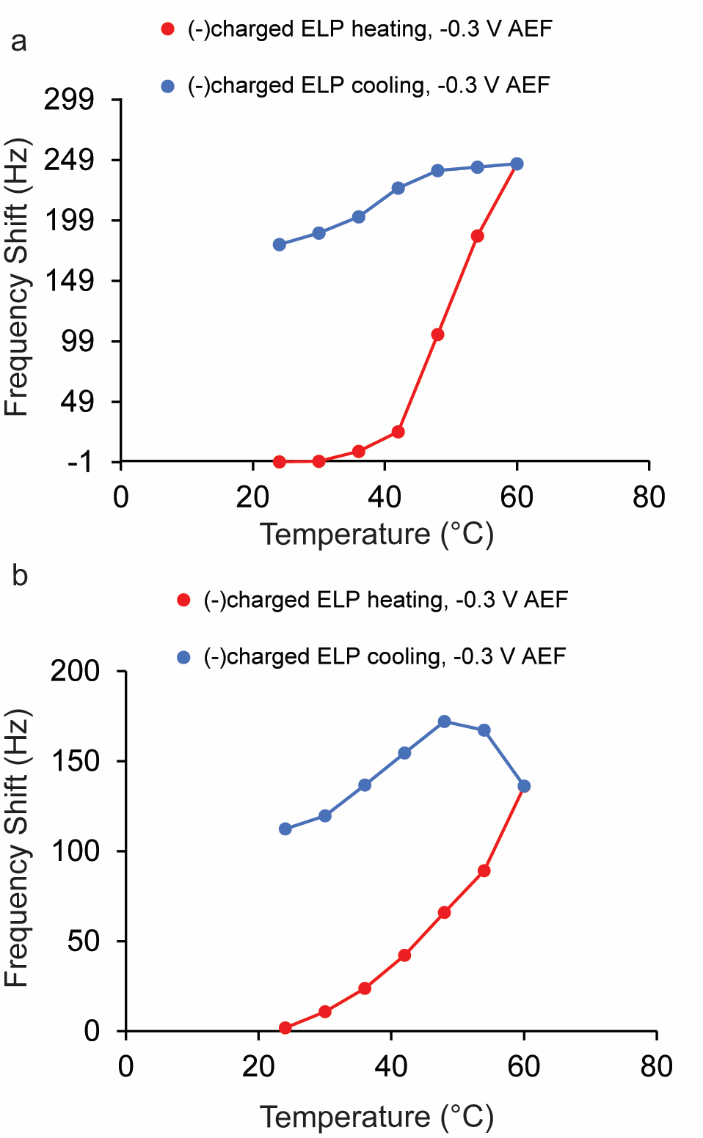


Fig. S10 Repeat experiments (a and b) of the surface-bound transition behavior of (-)charged ELP assembled in -0.3 V AEF vs. Ag/AgCl with heating (red) and cooling (blue). Lines between data points are added to help the reader more clearly see heating and cooling trends.


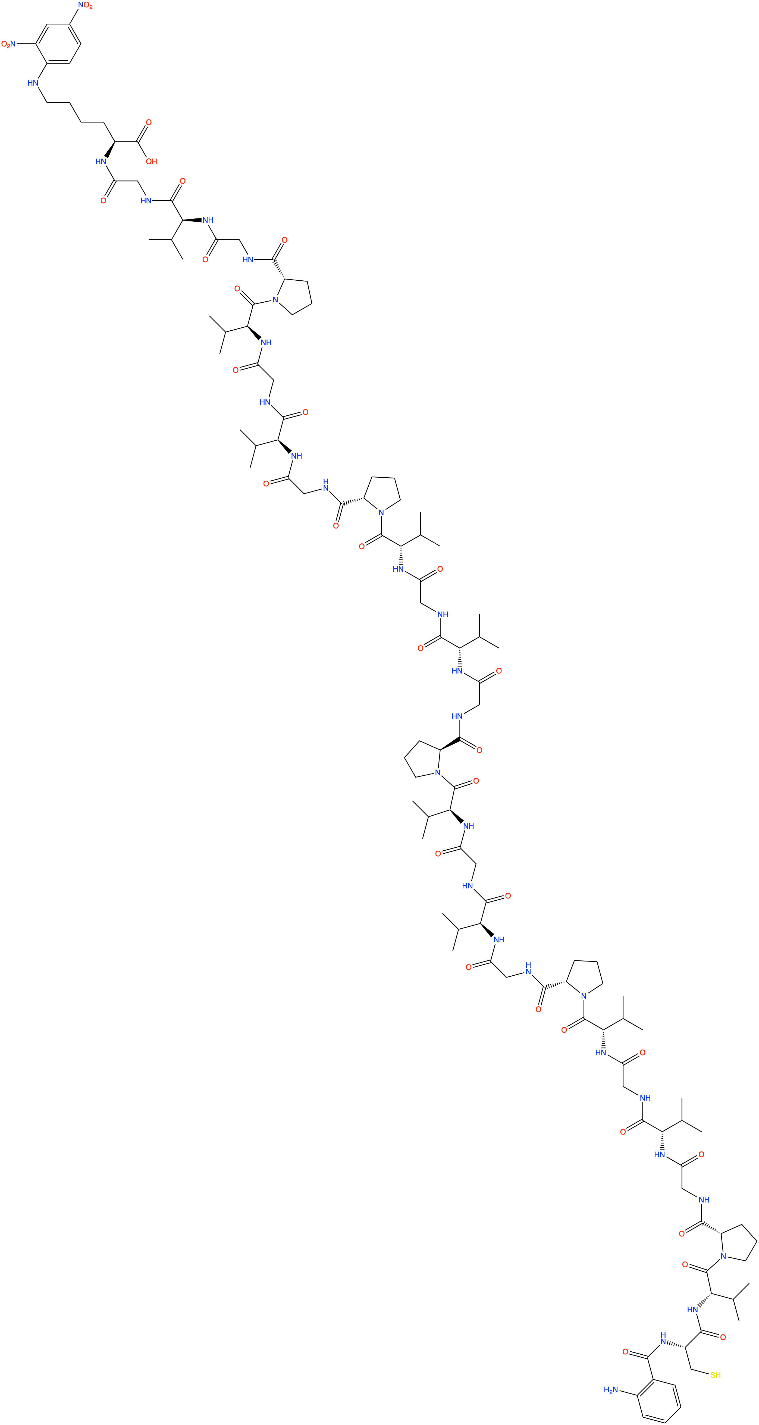


**Fig. S11** Negatively charged ELP chemical structure created in ChemDraw.

**References:**

1. Chen, Z., Ding, Z., Zhang, G., Tian, L. & Zhang, X. Construction of Thermo-Responsive Elastin-Like Polypeptides (ELPs)-Aggregation-Induced-Emission (AIE) Conjugates for Temperature Sensing. *Molecules* **23**, 1725 (2018).

2. Vu, C. Q., Fukushima, S. ichi, Wazawa, T. & Nagai, T. A highly-sensitive genetically encoded temperature indicator exploiting a temperature-responsive elastin-like polypeptide. *Scientific Reports* **11**, 1–14 (2021).

3. Urry, D. W. Physical Chemistry of Biological Free Energy Transduction As Demonstrated by Elastic Protein-Based Polymers ^†^. *The Journal of Physical Chemistry B* **101**, 11007–11028 (1997).

4. Meyer, D. E. & Chilkoti, A. Quantification of the Effects of Chain Length and Concentration on the Thermal Behavior of Elastin-like Polypeptides. *Biomacromolecules* **5**, 846–851 (2004).

5. Reguera, J., Urry, D. W., Parker, T. M., McPherson, D. T. & Rodríguez-Cabello, J. C. Effect of NaCl on the exothermic and endothermic components of the inverse temperature transition of a model elastin-like polymer. *Biomacromolecules* **8**, 354–358 (2007).

6. Cho, Y. *et al.* Effects of hofmeister anions on the phase transition temperature of elastin-like polypeptides. *Journal of Physical Chemistry B* **112**, 13765–13771 (2008).

7. Urry, D. W. *et al.* Temperature of polypeptide inverse temperature transition depends on mean residue hydrophobicity. *Journal of the American Chemical Society* **113**, 4346–4348 (1991).

8. McDaniel, J. R., Radford, D. C. & Chilkoti, A. A unified model for de novo design of elastin-like polypeptides with tunable inverse transition temperatures. *Biomacromolecules* **14**, 2866–2872 (2013).

9. Nicolini, C., Ravindra, R., Ludolph, B. & Winter, R. Characterization of the Temperature- and Pressure-Induced Inverse and Reentrant Transition of the Minimum Elastin-Like Polypeptide GVG(VPGVG) by DSC, PPC, CD, and FT-IR Spectroscopy. *Biophysical Journal* **86**, 1385–1392 (2004).

10. Schreiner, E. *et al.* Folding and Unfolding of an Elastinlike Oligopeptide: “Inverse Temperature Transition,” Reentrance, and Hydrogen-Bond Dynamics. *Physical Review Letters* **92**, 148101 (2004).

11. Groß, P. C., Possart, W. & Zeppezauer, M. An Alternative Structure Model for the Polypentapeptide in Elastin. *Zeitschrift für Naturforschung C* **58**, 873–878 (2003).

12. Selig, O. *et al.* Temperature-Induced Collapse of Elastin-like Peptides Studied by 2DIR Spectroscopy. *The Journal of Physical Chemistry B* **122**, 8243–8254 (2018).

13. Serrano, V., Liu, W. & Franzen, S. An infrared spectroscopic study of the conformational transition of elastin-like polypeptides. *Biophysical Journal* **93**, 2429–2435 (2007).

14. April 2015, 30. Infrared spectroscopy. *RSC Education* https://edu.rsc.org/resources/infrared-spectroscopy/1299.article.
